# Mr. Chamberlaine's Caution to Apothecaries, &c.

**Published:** 1810-01

**Authors:** W. Chamberlaine

**Affiliations:** 29, Aylesbury-street, Clerkenwell


### *To the Editors of the Medical and Physical Journal.*

Gentlemen,

As there are many respectable practitioners who deal extensively in retail articles of Pharmacy, I request, through the medium of your useful and widely circulated Publication, to give this public caution to the profession, that several druggists, and other vendors of medicines, have been served, at the suit of the attorney-general and Stamp-Office, with exchequer writs, for selling soda-water without stamps.

It will also be prudent for those who retail lozenges, to be very cautious how they sell lozenges of any description, whether named, or not named, in the schedule of the Medicine Act of 1803, without stamps.

I am, &c.

W. CHAMBERLAINE.

